# Biological Meshes for Inguinal Hernia Repair – Review of the Literature

**DOI:** 10.3389/fsurg.2015.00048

**Published:** 2015-09-15

**Authors:** Ferdinand Köckerling, Nasra N. Alam, Sunil K. Narang, Ian R. Daniels, Neil J. Smart

**Affiliations:** ^1^Department of Surgery, Center for Minimally Invasive Surgery, Academic Teaching Hospital of Charité Medical School, Vivantes Hospital, Berlin, Germany; ^2^Exeter Surgical Health Services Research Unit (HeSRU), Royal Devon and Exeter Hospital, Exeter, UK

**Keywords:** biological mesh, inguinal hernia, contaminated field, recurrence, pain

## Abstract

**Introduction:**

Biological meshes are a potential alternative to the synthetic meshes to avoid complications and are used in a contaminated field for incarcerated inguinal hernias. The clinical experiences gained with biological meshes for repair of inguinal hernias are presented in this review.

**Materials and methods:**

In a literature search of the Medline database using the key word “Biological mesh,” 2,277 citations were found. There remained 14 studies in which biological meshes had been used to repair inguinal hernias.

**Results:**

In prospective randomized trials, the use of polypropylene vs. biological meshes was compared in open inguinal hernia repair. There was no difference in the recurrence rate, but differences were observed in the postsurgical pain incidence in favor of the biological mesh. In the remaining retrospective studies, the recurrence rates were also acceptable. The biological mesh was used successfully in a potentially contaminated setting.

**Conclusion:**

Inguinal hernias can be repaired with biological meshes with reasonable recurrence rate, also as an alternative in a potentially contaminated field.

## Introduction

The Guidelines of the European Hernia Society state, based on evidence level 1 A, that operation techniques using mesh result in fewer recurrences than techniques, which do not use mesh (1). Although mesh repair appears to reduce the likelihood of chronic groin pain rather than increase it (1), mesh can cause considerable pain and stiffness around the groin and affect physical functioning (2). This has led to various types of mesh being engineered, with a growing interest in lighter weight polypropylene (PP) meshes (2), absorbable meshes (3), and biological meshes. For open inguinal hernia repair the use of light-weight PP meshes was not associated with an increased risk of hernia recurrence. Light-weight PP meshes reduce the incidence of chronic groin pain as well as the risk of developing other groin symptoms (4). To avoid complications, the use of absorbable meshes – such as those made of lactic acid polymer or lactic and glycolic acid copolymers – has been proposed. This exposes the patient to inevitable hernia recurrence because the inflammatory response, through a hydrolytic reaction, completely digests the implanted prosthetic material (3, 5).

Another potential alternative to the synthetic meshes is biological meshes which, unlike absorbable meshes, are not completely degraded; instead, these induce a remodeling process, i.e., the biological mesh is incorporated into the host through the reproduction of new site-specific tissue. The clinical experiences gained with biological meshes for repair of inguinal hernias are presented below.

## Materials and Methods

A literature search of the Medline database was performed using the PubMed search engine. The following key words were used: Biological mesh; inguinal hernia OR Groin hernia AND Biological mesh OR Biomesh OR Biological. 2,277 citations were found. After checking the title and abstracts, there remained seven prospective randomized trials (RCTs) (5–11). In one of these seven RCTs (Table 1), the results were reported for a smaller sample size (6) from the entire study (5) at an earlier follow-up time point. For two RCTs, only an abstract is available (8, 9). Recently, two meta-analyses were also published reporting on three and four RCTs, respectively (12, 13). Furthermore, there are five retrospective case series available (14–18), in which biological meshes had been used to repair inguinal hernias and the corresponding follow-up results reported (Table 2). These are also described below.

**Table 1:** **Characteristics and outcomes of RCTs on inguinal hernia repair with the use of biologic vs. polypropylene mesh**.

Reference	Study design	Patients characteristic	Mesh material	Intervention details	Follow-up	Outcome	Conflict of interest	LoE
(8) Abstract only	Prospective blinded randomized trial	*n* = 140 primary inguinal hernias	Collagen mesh vs. polypropylene	Open procedures	12 months	One recurrence in each group	NR	1b
(6)	Prospective double-blinded randomized trial	*n* = 20 primary inguinal hernias	SIS vs. polypropylene	Lichtenstein in general or spinal anesthesia	6 months	No recurrence, no wound infection, no post-hemioplasty acute and chronic pain/discomfort, parenteral/oral analgesic consumption were lower in surgisis group	NR	1b
(7)	Prospective randomized trial	*n* = 45 male patients with inguinal hernia	SIS vs. polypropylene vs. polylactic and polypropylene	Lichtenstein in local anesthesia	Mean: 12 months (1–16)	No recurrence, postoperative pain lower with SIS, full recovery shorter with SIS	NR	1b
(9) Abstract only	Prospective blinded randomized trial	*n* = 201	Porcine dermal collagen vs. polypropylene	Open procedure	24 months	No difference in recurrence rate, collagen repairs had improved pain scores	NR	1b
(5)	Double-blinded RCT	*n* = 70 primary inguinal hernia	SIS vs. polypropylene	Lichtenstein in general or spinal anesthesia	36 months	One recurrence in the PP group; significant lower pain degree for the SIS group	NR	1b
(10)	Prospective, double-blinded, single-center randomized trial	*n* = 100	Biodesign Inguinal Hernia Matrix (IHM) vs. polypropylene	Lichtenstein in local anesthesia	12 months	Three recurrences in the IHM group vs. 0 in the polypropylene group (*p* = 0.11). Persistent pain trended higher in the polypropylene group	Grant from producer of IHM	1b
(11)	Prospective, double-blinded, multicenter randomized trial	*n* = 172	Strattice vs. Ultrapro	Lichtenstein in local or general anesthesia	3 months	No recurrence, no wound complication, impairment caused by the hernia decreased significantly in both groups, less postoperative pain days 1 and 3 in the Strattice group	Grant form producer of Strattice	1b

**Table 2:** **Characteristics and outcomes of studies reporting on inguinal hernia repair with the use of biologic mesh**.

Reference	Study design	Patients characteristic	Mesh material	Intervention details	Follow-up	Outcome	Conflict of interest	LoE
(14)	Retrospective case series	*n* = 137 male patients *n* = 16 emergency cases	Porcine dermis (Zenoderm)	Modified Notaras-technique	Mean: 48 months	Two recurrences (1.25%)	NR	4
(18)	Retrospective case series	*n* = 15 potentially or grossly contaminated field	SIS	Laparoscopic TAPP	Median: 19 months (1–30)	No recurrence	NR	4
(17)	Retrospective case series	*n* = 10 sports hernia. Professional or amateur athletes	SIS	TEP; 7 cm × 10 cm mesh size, fixation with five tacks (Protack), one patient had only fibrin glue fixation	12 months	Nine improved, one not	NR	4
(15)	Retrospective case series	*n* = 38 patients with 45 primary and 6 recurrent inguinal hernias	SIS	TEP; 7 cm × 10 cm mesh size, fibrin glue fixation	Mean: 13 months (1–30)	One recurrence (2%), three patients chronic pain (7.9%)	NR	4
(16)	Retrospective case series	*n* = 11	SIS	TAPP; Fibrin glue fixation	Mean: 14.5 ± 1 month	One recurrence	NR	4

## Results

In a prospective randomized double-blind trial (5, 6), Lichtenstein’s inguinal hernia repair was compared using a PP or a small intestine submucosa (SIS) mesh. Seventy male patients underwent Lichtenstein’s hernioplasty, with 35 patients in the SIS group and 35 patients in the PP group. At 3 years after surgery, there were two deaths (5.7%) in the PP group and one death (2.9%) in the SIS group (NS). Only one recurrence (2.9%) was seen in the PP group (NS). Although a significant decrease in the postsurgical pain incidence was never observed among patients in the SIS group, a significantly lower degree of pain was detected at rest and on coughing at 1, 3, and 6 months and on movement at 1, 3, and 6 months and 1, 2, and 3 years. A significant decrease in the postsurgical incidence and degree of discomfort when coughing and moving were observed among patients in the SIS group at 3 and 6 months and at 1, 2, and 3 years after surgery. The authors concluded that SIS hernioplasty seems to be a safe and effective procedure.

In a prospective RCT (7), Lichtenstein inguinal hernioplasty was performed in local anesthesia, using prolene (PP) or vypro (polylactin and PP) or SIS. The median follow-up was 12 months, with a range of 1–16 months. No recurrent hernias were observed. Postoperative pain (visual analog scale) and discomfort were lower in patients with SIS. There was a tendency toward a higher incidence of pain and discomfort in the vypro and prolene group.

In an abstract as interim report, Macklin et al. (8) have treated 140 patients in a prospective RCT receiving either PP or collagen mesh. Postoperatively, there was an increase in hematoma in the PP group (*p* = 0.048). Infection and inflammation were similar postoperatively and at 3 months. There was one recurrent hernia in each group in 1 year.

Initial results showed that collagen mesh is an effective method of providing tissue repair in primary inguinal hernia.

In another abstract, Ridgway et al. (9) reported on a blinded randomized controlled trial comparing porcine dermal collagen with PP for primary inguinal hernia repair in 201 patients. Recurrence, inflammation, infection, and hematoma rates were comparable at all time intervals. Collagen repairs had improved pain scores at 2 years. The authors concluded that inguinal hernia repair using modified porcine dermal collagen can be performed successfully.

In another prospective, randomized, double-blinded, single-center study (10), the use of a Biodesign Inguinal Hernia Matrix (IHM) vs. a PP mesh for Lichtenstein operation was compared for 100 patients. The follow-up period was 1 year. Three recurrences were observed in the IHM group and none in the PP group (*p* = 0.11). There was a higher tendency toward persistent pain in the PP group (6 vs. 4%).

Likewise, in a prospective randomized, double-blinded multicenter study (11) that compared the use of Strattice vs. Ultrapro for Lichtenstein operation in 100 patients, no differences were observed in the wound complication rate after 3 months. No recurrences occurred in any of the two groups, nor any difference was seen in postoperative pain after 3 months.

On pooling, the results of the three (5, 7, 10) aforementioned RCTs, each of which used small intestinal submucosa (SIS), no difference was found in the recurrence and pain rate after 1 year (12). Only the discomfort rate was lower in the SIS group, but the seroma rate was higher. Likewise, these findings are confirmed in the meta-analysis of four (5, 7, 10, 11) RCTs (13).

In a retrospective case series Holl-Allen (14) published the results of 137 consecutive unselected male patients with inguinal hernias treated with Zenoderm as the repair material after a mean follow-up of 48 months. There have been two indirect recurrences after 11 and 14 months, representing a low recurrence rate of 1.25%.

In three retrospective case series (15–18) with 10–38 patients, inguinal hernias were repaired in an endoscopic technique (TEP, TAPP) with SIS. During a mean follow-up period of 12–14.5 months, a recurrence rate of 2 and 9.1% was observed, respectively (15, 16). No improvement in symptoms was seen in one patient with a sports hernia following TEP operation with SIS (17). In another study the biological meshes (SIS) were used successfully even in a potentially contaminated setting, i.e., with incarcerated/strangulated bowel within the hernia or coincident with a laparoscopic cholecystectomy/colectomy as well as in a grossly contaminated field (i.e., gross pus or fecal spillage) (18).

## Discussion

Inguinal hernias can be repaired with biological meshes, and with a reasonable recurrence rate. This applies for a period of 3 years for the Lichtenstein operation and of 1 year for the endoscopic TEP and TAPP techniques. As such, biological meshes can be used as an alternative in a potentially contaminated field for incarcerated inguinal hernia or coincident with a laparoscopic cholecystectomy or colectomy as well as in a setting grossly contaminated with pus or fecal spillage (18). However, this was a retrospective case series rather than a RCT. The RCTs identified demonstrated the equivalence of a biological mesh and the PP mesh in terms of the recurrence rate as well as reduced pain at rest, on coughing or on movement. Because of the very small sample size, the equivalence of biological meshes and synthetic meshes with regard to recurrence rate and reduced pain must be verified in further studies. Besides, in none of the studies were the higher costs incurred for the biological meshes analyzed. Since the biological meshes do not have any major advantages over the synthetic meshes with respect to the most important assessment criteria, at present they can only be recommended for situations involving a contaminated surgical field.

## Conflict of Interest Statement

The authors declare that the research was conducted in the absence of any commercial or financial relationships that could be construed as a potential conflict of interest.

## Appendix

### BioMesh Study Group

Ferdinand Köckerling (Chairman), Stavros Antoniou, René Fortelny, Frank A. Granderath, Markus Heiss, Franz Mayer, Marc Miserez, Agneta Montgomery, Salvador Morales-Conde, Filip Muysoms, Alexander Petter-Puchner, Rudolph Pointner, Neil Smart, Marciej Smietanski, and Bernd Stechemesser.

### Aim

The BioMesh Study Group has set itself the task of identifying how best to use biological meshes for the various indications. The first step toward achieving that goal is to compile systematic reviews of the different indications on the basis of the existing literature. The available literature sources will be evaluated in accordance with the Oxford Centre for Evidence-based Medicine-Levels of Evidence (March 2009). Next, based on the review findings corresponding Statements and Recommendations are to be formulated in a Consensus Conference for the use of biological meshes for the different indications. The findings of the Consensus Conference are then to be summarized for a joint publication. This present publication is part of the project undertaken by the BioMesh Study Group.
